# Surgical offloading procedures for diabetic foot ulcers compared to best non-surgical treatment: a study protocol for a randomized controlled trial

**DOI:** 10.1186/s13047-018-0248-3

**Published:** 2018-02-20

**Authors:** Aharon S. Finestone, Eran Tamir, Guy Ron, Itay Wiser, Gabriel Agar

**Affiliations:** 1 Department of Orthopaedic Surgery, Assaf HaRofeh Medical Center, Zerrifin, Affiliated to the Sackler School of Medicine, Tel Aviv University, POB 1424, Reut, 7179902 Tel Aviv, Israel; 2 Maccabi Health Services, Tel Aviv, Israel; 3 Department of Plastic Surgery, Assaf HaRofeh Medical Center, Tel Aviv, Israel; 4 Department of Epidemiology and Preventive Medicine, Sackler Faculty of Medicine, Tel-Aviv University, Tel Aviv, Israel

**Keywords:** Diabetic foot ulcers, Surgical offloading, Minimally invasive surgery

## Abstract

**Background:**

Diabetic foot ulcers are frequently related to elevated pressure under a bony prominence. Conservative treatment includes offloading with orthopaedic shoes and custom made orthotics or plaster casts. While casting in plaster is usually effective in achieving primary closure of foot ulcers, recurrence rates are high. Minimally invasive surgical offloading that includes correction of foot deformities has good short and long term results. The surgery alleviates the pressure under the bony prominence, thus enabling prompt ulcer healing, negating the patient’s dependence on expensive shoes and orthotics, with a lower chance of recurrence. The purpose of this protocol is to compare offloading surgery (percutaneous flexor tenotomy, mini-invasive floating metatarsal osteotomy or Keller arthroplasty) to non-surgical treatment for patients with diabetic foot ulcers in a semi-crossover designed RCT.

**Methods:**

One hundred patients with diabetic neuropathy related foot ulcers (tip of toe ulcers, ulcers under metatarsal heads and ulcers under the hallux interphalangeal joint) will be randomized (2:3) to a surgical offloading procedure or best available non-surgical treatment. Group 1 (surgery) will have surgery within 1 week. Group 2 (controls) will be prescribed an offloading cast applied for up to 12 weeks (based on clinical considerations). Following successful offloading treatment (ulcer closure with complete epithelization) patients will be prescribed orthopaedic shoes and custom made orthotics. If offloading by cast for at least 6 weeks fails, or the ulcer recurs, patients will be offered surgical offloading. Follow-up will take place till 2 years following randomization. Outcome criteria will be time to healing of the primary ulcer (complete epithelization), time to healing of surgical wound, recurrence of ulcer, time to recurrence and complications.

**Discussion:**

The high recurrence rate of foot ulcers and their dire consequences justify attempts to find better solutions than the non-surgical options available at present. To promote surgery, RCT level evidence of efficacy is necessary.

**Trial registration:**

Israel MOH_2017–08-10_000719. NIH: NCT03414216.

## Background

Pressure ulcers are common complications in patients with peripheral neuropathy. Most peripheral neuropathy nowadays is related to diabetes mellitus (DM), and can be found in up to 67% of patients with type 2 DM [1]. The annual incidence of ulcers in patients with DM is about 2% [2] with global prevalence of diabetic foot ulcers as high as 6.3% [3] and ulcers having been implicated as a causative factor in up to 84% of diabetic foot amputations [4]. The estimated annual cost of diabetic foot ulcers (DFU) in the United States is $9 billion to $13 billion [5, 6]. In the presence of sensory neuropathy and lack of protective sensation, an ulcer can develop in a foot with normal anatomy as result of an acute injury. But more frequently, abnormal pressure develops because of an anatomical deformity in the foot, frequently resulting from long standing muscular imbalance related to the neuropathy itself [7], even though this relationship is not straightforward [8]. The mainstay of treating and preventing ulcers is offloading. This may be done with shoes, orthotics and contact casts [9–11]. But while these are frequently effective in the short run, in the long run ulcers often recur for a variety of reasons, including patients’ lack of compliance. Pound et al. reported a 40% recurrence rate within a mean of 126 days [12]. Armstrong et al. estimated 3 year recurrence rates to be almost 60% [13]. A more definitive method of offloading includes surgical correction of foot deformities. While any surgery in these patients is a considerable undertaking, the natural history of recurrent or recalcitrant ulcers is so dismal that a more aggressive and surgical approach may be justified. Newer minimally invasive surgical techniques have the potential to lower previously deterrent high complication rates. Retrospective data seem to support flexor tenotomies for tip of toe ulcers [14–22]. In a randomized control trial (RCT) comparing Achilles tendon lengthening with usage of total contact cast to usage of the cast alone in patients with neuropathic plantar ulceration, the recurrence of the ulcers in the first group was significantly lower than in the second group at 7 months follow up (15% vs. 59%) and at 2 years (38% vs. 81%) [23]. In this study, the procedure had negative implications on the foot biomechanics and possibly a slight decrease in overall function as assessed by SF36 [24, 25]. Another RCT compared the effect of debridement, removal of bone segments underlying the lesions and surgical closure compared with conservative treatment for offloading diabetic ulcers [26]. The results showed a significantly lower recurrence rate in the first group at 6 months follow up. These have been the only types of operation supported by RCT level data, and there is very limited prospective data [27, 28]. A recent systematic review by the International Working Group on the Diabetic Foot summarized: "no definitive statements can yet be made regarding the efficacy and safety of surgical interventions to heal foot ulcers or to prevent recurrence, because of the limited number of RCTs" [11].

Our team at Assaf HaRofeh has recently published successful results for several offloading surgical procedures [15, 18, 29, 30] as have other clinicians with other procedures [31]. Our standard management included clinical assessment of the foot deformity before and after surgery to see how well the deformity had been corrected. We also noted the clinical results: improvement and in most cases healing of the ulcer and disappearance of the callosities that had developed at sites of pressure, within 2–3 weeks. The three procedures that are subject of this proposal are:

1. Percutaneous flexor tenotomy for tip of toe ulcers [15, 17].
2. Minimally invasive floating metatarsal osteotomy for pressure ulcers under the metatarsal heads 1–5 [30].
3. A modified Keller resection arthroplasty of the 1st metatarsophalangeal joint for ulcers under the hallux [29].

The purpose of this open randomized controlled semi-crossover designed trial is to compare the efficacy of surgical offloading procedures to best available non-surgical treatment in curing diabetic foot pressure ulcers and in preventing recurrence within 2 years.

## Methods

Study design is according to CONSORT guidelines. Patients with a Texas stage A, grade 1 or 2 diabetic-neuropathic ulcer attributable to an anatomical deformity, examined at a foot and ankle outpatient clinic specializing in the treatment of diabetic foot ulcers will be approached to take part in a semi-crossover designed RCT assessing the efficacy of the surgery in healing the ulcer and preventing its recurrence. Specific indications include: 1) tip of toe ulcer related to hammer or claw toe, 2) ulcer under metatarsal head related to low-riding metatarsal head & 3) ulcer under the interphalangeal joint, related to “functional hallux limitus” [32] with high pressure under the hallux. The first 100 patients to consent will answer basic questionnaires on the diabetes, feet, ulcer and concurrent illnesses. Baseline data will include age, sex, ethnicity (Jewish categories: European/American, North African, Persian Gulf, Yemenite, Ethiopia, mixed. Non-Jewish categories: Arab, other), ulcer duration, history of previous ulcer, diabetes type and duration, duration of insulin treatment, comorbidities including complications of diabetes and others, and ambulatory status. Specific physical examination will include ulcer site, length, width, depth, probe-to-bone, redness surrounding the ulcer, discharge, palpation of dorsal pedal & tibialis posterior pulses, Ankle Brachial Index (ABI, with Doppler assessment in the absence of pulses orABI< 0.9) and 5.07/10 g monofilament sensory test [33–35]. Laboratory tests will include a blood count, HbA1c & creatinine. Plantar foot pressures will be recorded before surgery and during follow-up on a MatScan plate (Pressure mapping sensor 3150, Tekscan, Boston MA).

*Group 1 (surgery)* will be operated on within 1 week.

*Group 2 (controls - best available non-surgical treatment)* will be prescribed offloading in a fiberglass offloading cast (applied by a trained & experienced technician). Patients that fail cast treatment (due to complications or compliance) will be treated with a full length, padded, removable walking boot (e.g. the Aircast XP diabetic walker™ - http://www.djoglobal.com/products/aircast/xp-diabetic-walker-system) or a healing shoe (e.g. Darco OrthoWedge™ - http://www.darcointernational.com/orthowedge). Adherence to treatment will be monitored by a questionnaire filled in at follow-up visits detailing various activities such as going to bathroom at night. Objective measures of compliance to treatment such as the orthotimer (http://www.orthotimer.com) will be utilized where the technology is available. Non-surgical treatment will be continued until the ulcer heals or up to 12 weeks. Primary assessment will be at 6 weeks. If the ulcer is improving, further cast treatment will be recommended, up to 12 weeks. If the ulcer is not healing with non-surgical treatment, or has not healed completely at 12 weeks, the patient will be offered surgery. Crossover to surgery will not be permitted before 6 weeks of non-surgical treatment. When the ulcer heals (complete epithelization) the cast will be replaced by orthopaedic shoes and custom made offloading insoles.

**Fig. 1:**
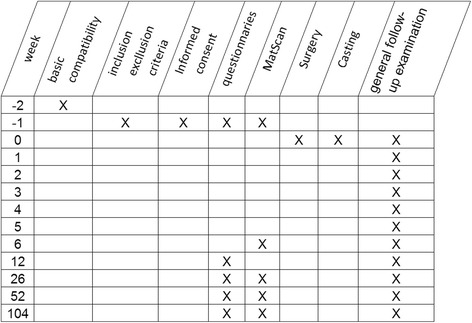
Treatment Flowchart - Time Schedule. Note that crossover patients will start afresh from the beginning

*Follow-up* will take place at weeks 1, 2, 4 and 6 after surgery, and weekly during non-surgical treatment up to complete wound closure or up to 12 weeks. Further follow-up will be at 3, 6, 12, 18 & 24 months following treatment. Follow-up will include questionnaires, physical examination and repeated plantar foot pressure measurements (Fig. 1). For patients treated with orthotics, pressure alleviation will be monitored clinically, with in-shoe plantar pressure measurements if the technology is available.

### Inclusion criteria

Consenting adult patients with a Texas stage A, grade 1 or 2 diabetic-neuropathic ulcer in the tip of a toe, under a metatarsal head or under the big toe, ulcer attributable to an anatomical deformity (hammer or claw toe, low-riding metatarsal head, high pressure under the hallux, respectively).

ABI > = 0.9 with palpable pulses or a duplex scan that demonstrates bi/triphasic pulses to 2 vessels at the level of the ankle [36].

### Exclusion criteria

Not able to understand the language of the informed consent form, not likely to be compliant with the protocol, infection, ischaemia of the limb, more than one ulcer in the assigned foot (with the exception of tip of lesser toe ulcers with no other ulcers).

Criteria for experiment cessation: A safety board (2 senior orthopaedists and 1 internal medicine specialist, without conflict of interest) will review all serious adverse events (SAE’s). An SAE rate of more than 20% in the first 20 subjects or above 10% in the following subjects should be of concern. SAE’s shall be defined according to the FDA ICH (life threatening, death, hospitalization/prolongation of hospitalization, persistent or significant disability/incapacity, required intervention to prevent permanent impairment/damage). The reasoning for the stated rates is that these patients are at high risk for various complications without connection to the study.

### Criteria for participation cessation

Participant’s request. Reasoning: once allocation & primary treatment has taken place, all treatment is according to best known practice, and decisions will be clinical, and not dictated by the research protocol. If there is any doubt, the safety board will be consulted. If complications do occur, research level follow-up is mandatory, unless patient explicitly objects.

### Randomization

To ensure allocation concealment, permuted block randomization, stratified by procedure type, will be performed. Following informed consent signature, allocation will be given to the recruiting surgeon over the phone from an independent source. Patients will be randomized 40:60 (surgery:non-surgery, rationale for this is detailed in the discussion).

**Fig. 2:**
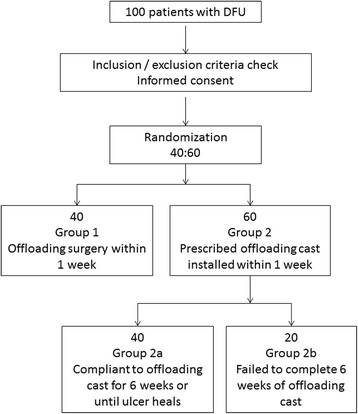
Protocol group flowchart

After the first 20 subjects are recruited and non-surgical subjects have completed the compulsory 6 weeks, if compliance is out of the 50–80% range the randomization will be repeated at a corrected ratio (Fig. 2).

### Surgical techniques

All bony procedures (excluding percutaneous tenotomies) will be given pre-operative antibiotics (2 g cefazolin IV) or a relevant substitute in case of hypersensitivity.

**Fig. 3:**
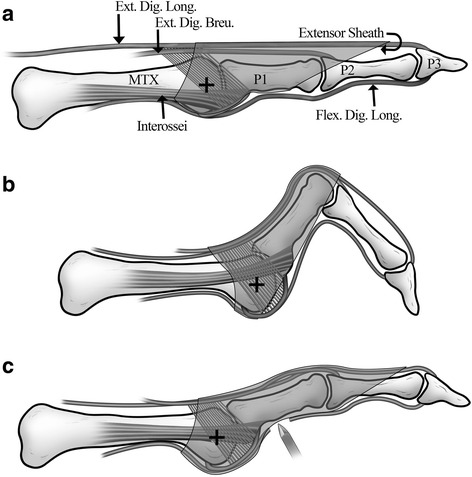
A schematic representation of the mechanism of tip of toe ulcer formation and treatment. **a** The normal toe. Note how the interosseii (and lumbricals, not delineated) pass below the center of the head of the metatarsal (marked with a cross) inserting into the extensor hood. They act as flexors of the metatarso-phalangeal joint and extensors of the proximal and distal inter-phalangeal joints [45].. **b** In absence of the flexing moment of the interosseii, the extensor digitorum longus forces the metatarso-phalangeal joint into extension. In absence of the extending moment of the interosseii and lumbricals through the extensor sheath, the flexor digitorum longus forces the proximal and distal inter-phalangeal joints into flexion. **c** The flexor tenotomy with the Beaver knife straightens the toe, relieving pressure from the ulcer sites. Reproduced with permission from Foot & Ankle International [15]

*Tip of toe ulcers* will be treated by percutaneous tenotomy [18]. The feet will be cleansed with alcohol chlorhexidine and the procedures will be performed with sterile gloves and a mask. A digital block will be performed with 5 cm^3^ of 1% lidocaine, except in patients with sensory neuropathy severe enough to make anesthesia unnecessary. For long flexor tenotomy, the tendon of the toe will be placed under tension by dorsiflexing the ankle and toe. The patient will be asked to actively flex the toe to cause bow stringing of the flexor tendon. A beaver blade (BB361, Aesculap, Germany) will be inserted at the midline of the base of the middle phalanx making a tiny puncture wound. The tendon will be gently cut by careful side-to-side micro movements of the blade tip (Fig. 3) without straying medially or laterally (to avoid injury to the neurovascular bundles). At the end of the procedure, inability of the patient to flex the distal interphalangeal joint will confirm that the tendon has been completely severed. The patients will be advised to rest and use a “post-operative” shoe for one week. At follow-up the compliance to the “post-operative” shoe will be questioned.

**Fig. 4:**
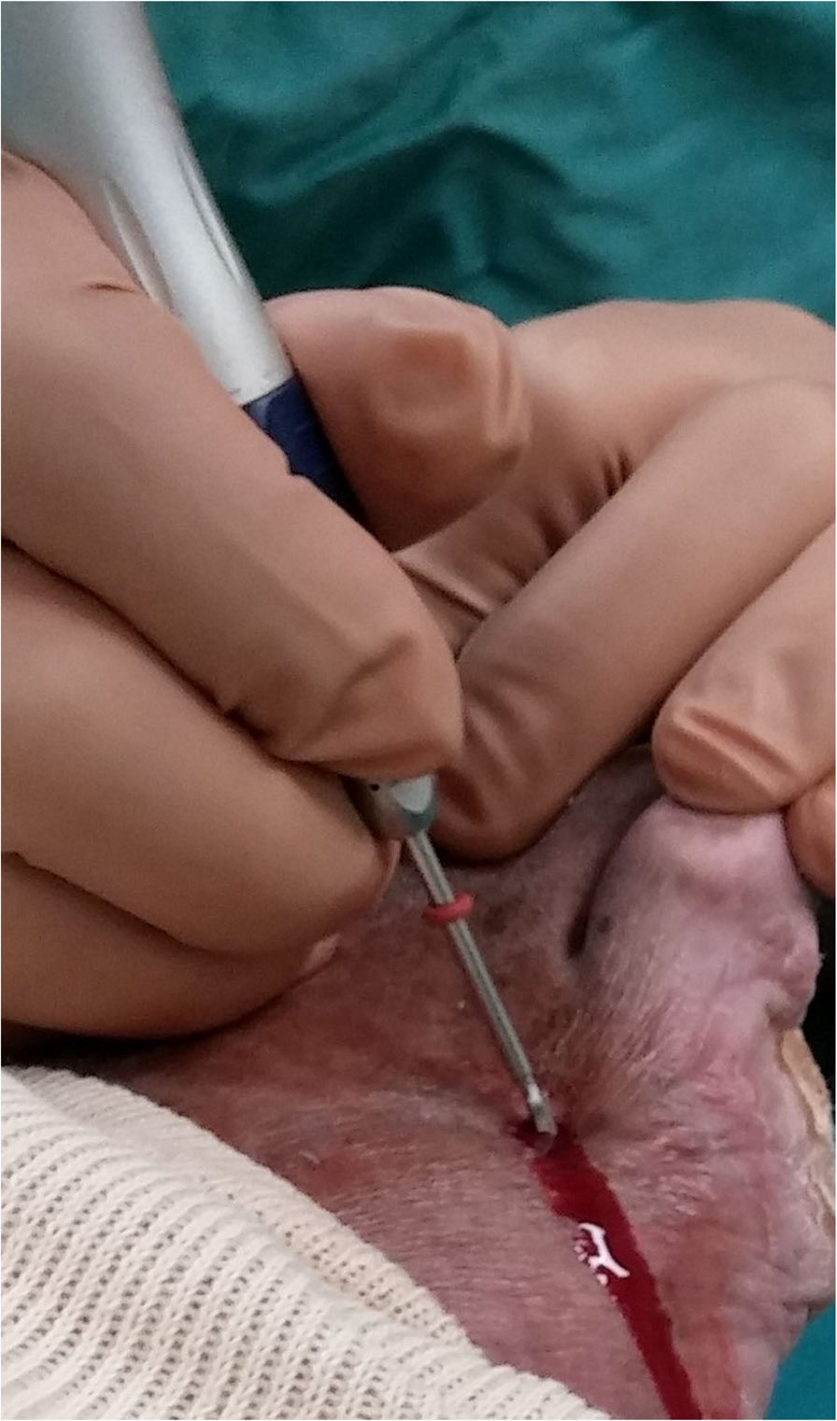
Minimally invasive floating metatarsal osteotomy. Surgical technique with Shannon burr

**Fig. 5:**
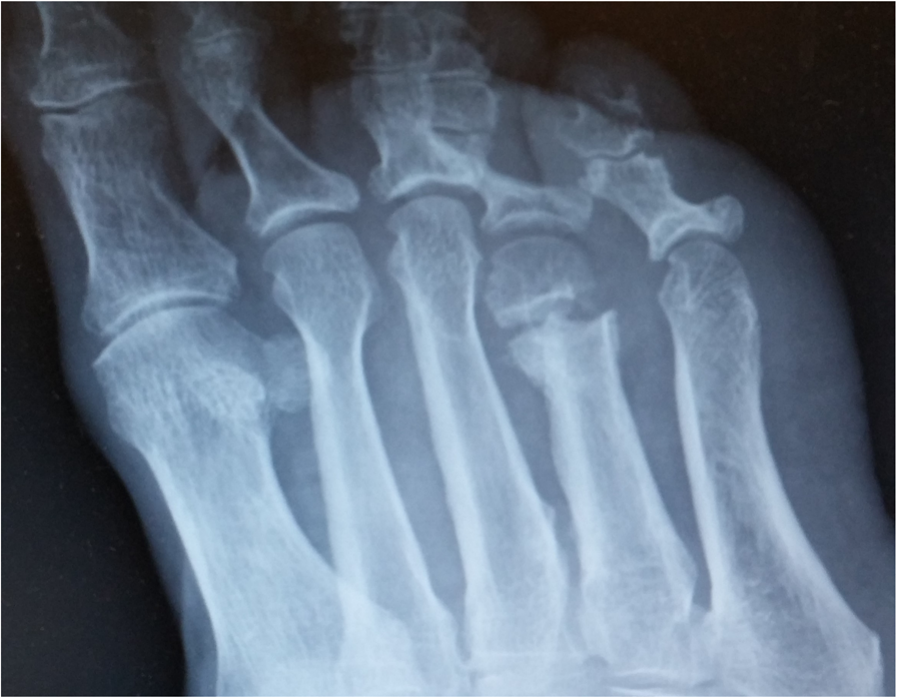
Minimally invasive floating metatarsal osteotomy. Post-operative x-ray demonstrating an osteotomy of the neck of the 4th metatarsal

*Ulcers under metatarsal heads* will be offloaded with a minimally invasive floating metatarsal osteotomy [30]. Anaesthesia by ankle block will be performed with 20cm^3^ of 1% lidocaine except in patients with neuropathy severe enough to make anesthesia unnecessary. Scrubbing and draping will be as standard for foot and ankle surgery. A 3 mm incision will be made dorsally at the planned osteotomy site after fluoroscopic identification (Figs. 4 and 5). A perpendicular or short oblique osteotomy will be made at the neck or diaphysis of the affected metatarsus (2–5) or metaphysis of metatarsus 1 (after Giannini [37] but without K-wire fixation) with a 12*2 mm Shannon burr at a speed of 1600 rounds per minute and a torque of 80 N-meter. Fluoroscopy will be used again to confirm completion of the osteotomy. Following the osteotomy, the metatarsal head will be displaced dorsally. No fixation will be used. Skin closure will be achieved with a single 4–0 nylon suture. Full weight bearing in a “post-operative” shoe will be permitted immediately. The shoe will be used for 4 weeks. At follow-up the compliance to the “post-operative” shoe will be questioned.

**Fig. 6:**
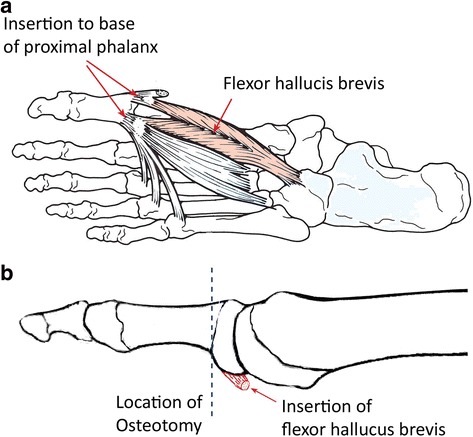
Schematic outline of Keller resection arthroplasty that includes shortening the toe by osteotomy of the proximal phalanx and detaching the flexor hallucis brevis tendon. Reproduced with permission from Foot & Ankle International [29]

*Ulcers plantar to the interphalangeal joint of the hallux* will be treated by a modified Keller resection arthroplasty (originally designed for the treatment of hallux valgus in otherwise healthy patients) [29]. Anaesthesia by ankle block will be performed with 20cm^3^ of 1% lidocaine except in patients with neuropathy severe enough to make anesthesia unnecessary. Scrubbing and draping will be as standard for foot and ankle surgery. Skin incision will be just medial to the extensor hallucis longus tendon. The joint capsule will be opened longitudinally and the joint exposed. A 4 to 5 mm slice of bone and cartilage will be cut from the base of the proximal phalanx perpendicular to the bone’s axis with a saw, detaching the short flexor (Fig. 6). The slice will be removed carefully, aiming to remove it in one piece if possible. A shallow cup shaped indentation will be created in the proximal first phalanx with a burr drill (creating a negative to the head of the first metatarsal) to increase the congruency with the metatarsal head, promote smoother movement, increase the toe shortening effect and facilitate forming a pseudo-arthrosis. The joint capsule will be sutured tightly and the wound will be closed in layers and the foot dressed. A non-weight bearing cast will be applied for 2 weeks. After cast removal patients will wear a “post-operative” shoe for another 2 weeks. Compliance to the “post-operative” shoe will be questioned at follow-up.

### Casting techniques

*Tip of toe ulcers* and *ulcers plantar to the interphalangeal joint of the big toe* will be casted in a fiberglass cast with a heel, ending under the metatarsal heads, leaving the toes in the air.

**Fig. 7:**
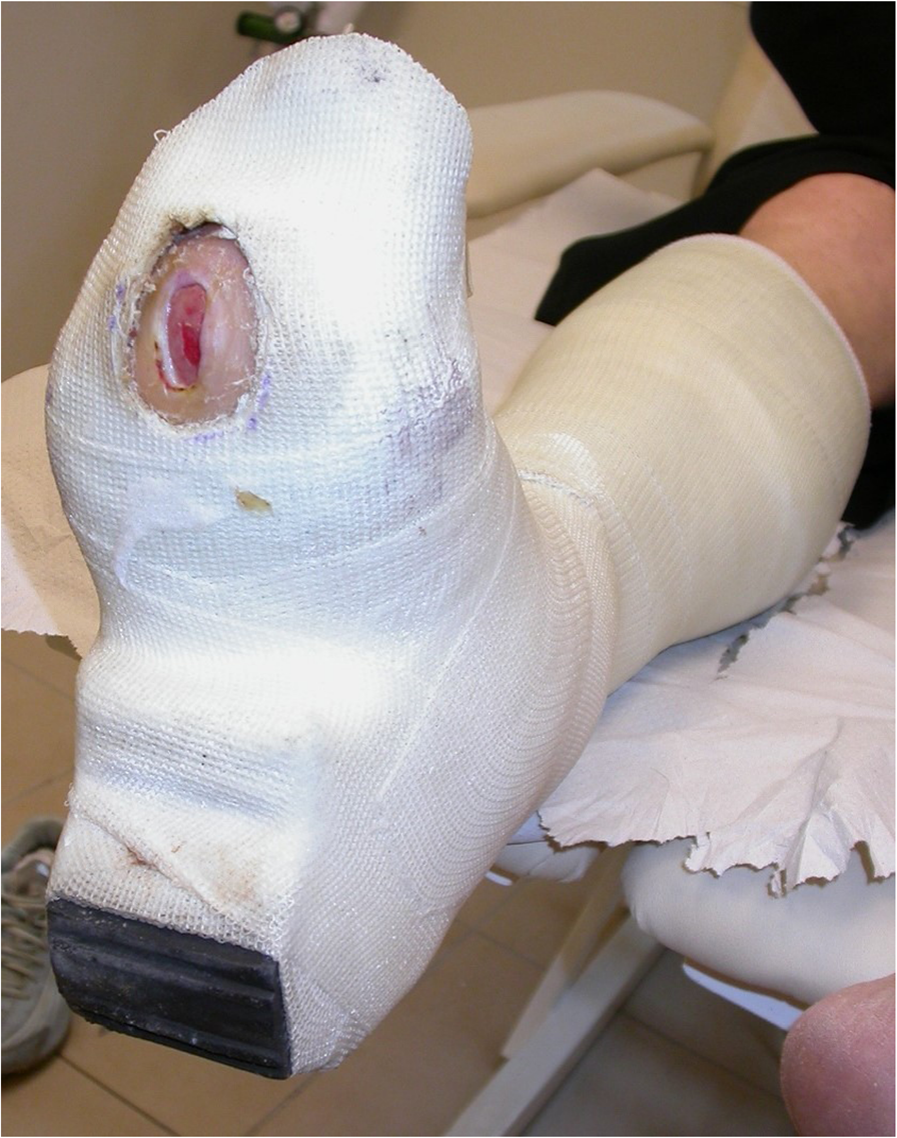
Fiberglass cast with heel for metatarsal head ulcers

*Ulcers under metatarsal heads* will be casted in a full foot fiberglass cast with a heel with a window below the ulcer designed to relieve pressure under the metatarsal heads and follow the ulcer (Fig. 7).

### Grouping

*Group 1* will include all patients randomized for surgery and operated on. Randomized patients in group 1 that decline surgery (post randomization) will be excluded from per-protocol analysis. *Group 2a* will include all patients randomized to cast offloading that completed at least 6 weeks of treatment or had complete ulcer healing. *Group 2b* will include patients randomized to cast offloading that failed to complete 6 weeks of cast offloading due to complications or lack of compliance.

### Outcome measures and analysis

The main outcome will be success or failure of treatment at 2 years. Success will be defined as complete healing (epithelization) at 12 weeks with no recurrence.

Failure will be defined as a composite of lack of complete closure at 12 weeks or recurrence within 2 years from surgery. Outcome measures will include time to ulcer healing (complete epithelization) time to surgical wound healing, ulcer length, width & depth, complications and recurrence. Primary (intention to treat) analysis of treatment success will be between 3 groups (1 versus 2a & 2b) where group 1 & group 2a are likely to have similar short term results and group 2b inferior results. Per protocol analysis will be between groups 1 & 2a. Recurrence will be compared between all patients whose ulcer healed (group 1 versus groups 2a & 2b). Total 2-year success rate will be calculated as the percentage of patients without ulcer and without recurrence (any recurrence of an ulcer at any time during the 2 years will count as recurrence). For survival analysis, the comparison will be between surgery (group 1 & patients in group 2 that crossed over, after the crossover) and group 2 (a & b, before the crossover, so crossover patients are included twice, once in each group).

### Statistics and sample size calculation

From previous studies, cure rate is likely to be about 90% in both group 1 and group 2a [9, 15, 29, 30]. Recurrence in group 1 is predicted to be no more than another 10%, bringing overall failure at 2 years to 20%. Recurrence in group 2a is likely to be around 50% [12, 38–40]. To compare 20% & 60% failure (initial & recurrence), α = 0.05, β = 0.2, we need 23 subjects in each group (total: 46). Time to ulcer healing and time to surgical wound healing will be compared using survival analysis (SAS: PROC LIFETEST) and Chi square. Complications and recurrence will be compared using Chi square. Our calculations are based on the clinical data in our clinic, different from those presented by Armstrong et al. e.g. for recurrence [13].

## Discussion

While preventive medicine is usually considered to be a superior approach to treating disease already manifested, little research has been invested in DFU prevention [41]. In diabetic patients prior to the first ulcer, how much to invest in prevention is a legitimate question as most patients will not develop ulcers [1]. But patients who already have an ulcer are immediately bounced into DFU risk group 3A with a 2 year risk of 50.5% for another ulcer and 36.3% for amputation [40]. These data are probably pessimistic for the patients we propose to include (primarily because we exclude peripheral vascular disease) but the challenge set by Bus & van Netten to prevent 75% of ulcers [41] will definitely need a more aggressive approach. We are not yet ready to present a study on surgical prevention. There does not yet seem to be enough data out there to justify an RCT on patients without ulcers. In our study we are offering surgery to treat an existing ulcer, and following recurrence.

Generally, surgical treatment has been subject of RCT’s to a lesser degree than pharmaceuticals. This is related to ethical issues, physician - patient issues and the fact that there is no formal demand for RCT level data before new surgical procedures are allowed to be introduced [42]. The high success rates of surgery both in curing and preventing ulcer recurrence demonstrated in retrospective studies, together with the dismal outlook of recurrence and complications using standard best care treatment make the surgical option seem reasonable [40]. But only RCT’s can give a reasonable amount of certainty to whether the surgical option is indeed advantageous.

During planning the control group, we encountered several problems. While offloading with an un-removable cast is clearly the best available medical practice [9, 43, 44] most patients with DFU’s (including in our clinic) are treated with less effective means, such as removable casts, healing shoes or orthopaedic shoes with orthotics. We considered having a control group with removable casts or healing shoes, a design that would probably increase the treatment effect, but in designing an RCT, this may not be ethical (offering the control group sub-optimal treatment). We therefore decided to offer all patients offloading casting, assuming there will be little treatment effect on healing (both groups will be adequately offloaded during the first few weeks), and the main measured effect in the compliant subjects will be recurrence rates.

A major practical consideration is the compliance rate in the control group. While we assume that following informed consent, there will not be much dropout of the surgery group, this is not the case for the controls. Beyond cast related inconvenience and complications (possibly counted as failures of the non-surgical treatment) some of the patients will not comply with the minimum 6 weeks of cast treatment before requesting to crossover to surgery. This is even more likely because the patients know about the surgical option, and have already decided to consent for surgery. It is obviously not possible to continue the cast treatment against a patient’s will. We will therefore abort the cast treatment in patients that so desire, and continue with other more comfortable offloading methods such as a removable walking boot or a healing shoe (the best treatment possible that they are agreeable to), to enable crossover to surgery after a minimum of 6 weeks of nonsurgical treatment (continuing full follow up). A 6 week wait for this type of elective surgery, for a problem that has usually been present for months, seems reasonable in most health care systems.

As we cannot know in advance the size of the noncompliant group, we will increase the size of the control group to 60%. Inevitably we will have 3 groups: group 1) surgical treatment, group 2a) casted till ulcer healed or at least 6 weeks, and group 2b) noncompliant to cast, cast removed before 6 weeks without complete ulcer healing with offloading continued by removable cast or with calcaneal healing shoe up to 6 weeks. A further problem of unknown magnitude is whether patients in the control group will pressure the surgeon for surgery. This issue seems resolved by the directive that crossover will not be permitted until the patient completes at least 6 weeks of adequate nonsurgical treatment, and this will be explained and documented in the informed consent statement.

A further important comment regards the surgical techniques. Those cited are based on our experience with our patients. Other clinicians have good results with their procedures (e.g. hallux interphalangeal arthroplasty for ulcers under the interphalangeal joint [31]). The innovation in this protocol is the semi-crossover design. We recommend implementing this protocol to test the procedures that each clinician is successful with.

## Abbreviations

ABI: ankle brachial index
DFU: diabetic foot ulcer
DM: diabetes mellitus
FDA: ICH Food & Drug Administration - International Council for Harmonisation
RCT: randomized controlled trial
